# Analysing the socioeconomic determinants of hypertension in South Africa: a structural equation modelling approach

**DOI:** 10.1186/1471-2458-14-414

**Published:** 2014-05-01

**Authors:** Annibale Cois, Rodney Ehrlich

**Affiliations:** 1 School of Public Health and Family Medicine, University of Cape Town, Anzio Road, Observatory 7925, Cape Town, South Africa

**Keywords:** Systolic blood pressure, Diastolic blood pressure, Hypertension, Body mass index, Socioeconomic status, Sub-Saharan Africa, Structural equation modelling

## Abstract

**Background:**

Epidemiological research has long observed a varying prevalence of hypertension across socioeconomic strata. However, patterns of association and underlying causal mechanisms are poorly understood in sub-Saharan Africa. Using education and income as indicators, we investigated the extent to which socioeconomic status is linked to blood pressure in the first wave of the National Income Dynamics Study — a South African longitudinal study of more than 15000 adults – and whether bio-behavioural risk factors mediate the association.

**Methods:**

In a cross-sectional analysis, structural equation modelling was employed to estimate the effect of socioeconomic status on systolic and diastolic blood pressure and to assess the role of a set of bio-behavioural risk factors in explaining the observed relationships.

**Results:**

After adjustment for age, race and antihypertensive treatment, higher education and income were independently associated with higher diastolic blood pressure in men. In women higher education predicted lower values of both diastolic and systolic blood pressure while higher income predicted lower systolic blood pressure. In both genders, body mass index was a strong mediator of an adverse indirect effect of socioeconomic status on blood pressure. Together with physical exercise, alcohol use, smoking and resting heart rate, body mass index therefore contributed substantially to mediation of the observed relationships in men. By contrast, in women unmeasured factors played a greater role.

**Conclusion:**

In countries undergoing epidemiological transition, effects of socioeconomic status on blood pressure may vary by gender. In women, factors other than those listed above may have substantial role in mediating the association and merit investigation.

## Background

Socioeconomic disparities in the prevalence of hypertension have long been observed in high income countries, where sound epidemiological evidence associates higher socioeconomic status (SES) with a lower prevalence of high blood pressure and cardiovascular disease, an association that is consistent across a variety of indicators of social position [1,2].

By contrast, the pattern of association appears diverse in sub-Saharan Africa (sSA), where a mix of positive and negative gradients has been found across studies, in some distinct by gender [3-6].

Inconsistencies in SES measurements, sample heterogeneity and different degrees of economic development have been argued as possible explanations of these conflicting results [7]. However, the overall picture is far from complete and a better understanding of the reasons for this heterogeneity is needed in order to inform population based preventive interventions. Such understanding requires going beyond simply describing the association between SES and hypertension to identifying potentially modifiable mediating factors and causal pathways though which socioeconomic factors affect blood pressure.

Modifiable bio-behavioural factors affecting blood pressure levels and risk of hypertension include body mass index (BMI) and other measures of body shape such as waist circumference, as well as resting heart rate, alcohol consumption, exercise, and smoking. A positive association between BMI and blood pressure has been consistently observed in a large number of studies, including in sSA [3,8-10]. Similarly, blood pressure tends to increase with alcohol consumption [11], and a positive relationship between resting heart rate and hypertension has been repeatedly found [12]. Conversely, physical activity is associated with lower blood pressure, and the relationship persists after adjustment for the body weight reduction associated with increased activity [13,14]. Despite the fact that acute effects of smoking result in a transient rise in blood pressure, the evidence of increased risk of hypertension among smokers is scarce [15], and most observational studies show, conversely, that habitual smokers have lower blood pressures than non-smokers [16,17].

These biological and behavioural factors are often unevenly distributed across socioeconomic strata, making them suitable candidates as mediators of the observed effects of socioeconomic variables on blood pressure [2]. Two recent studies have directly tested this hypothesis and analysed the extent to which the above factors mediate the association between SES and blood pressure [18,19]. Their findings suggest that variations in BMI/waist circumference, heart rate, smoking and alcohol use account for a sizable proportion of the socioeconomic inequalities in blood pressure levels. However, both studies were carried out in western countries with high level of income per capita, relatively efficient health systems and morbidity profiles largely characterised by chronic diseases, while equivalent studies in developing countries, such as those in sSA, are lacking.

Recently, the National Income Dynamics Study (NIDS) [20] made available good quality anthropometric, sociodemographic and behavioural data for a large sample of the South African population. The analysis of these data offers an opportunity to improve the understanding of the relationships between SES and hypertension in a middle income country undergoing a rapid and complex epidemiological transition [21], and whose morbidity profile comprises coexisting infectious diseases (including a widespread epidemic of HIV/AIDS, the leading cause of worsened mortality between 1990 and 2005), increasing rates of non-communicable diseases and risk factors for cardiovascular disease, persisting child diarrhoea and malnutrition, and interpersonal violence and accidents [22].

The aim of the present study was (1) to assess the independent association of education and income, as SES indicators, with blood pressure in the adult population of South Africa, and (2) to examine the extent to which differences in body mass, resting heart rate, smoking and alcohol use explain these relationships.

## Methods

### Participants

This study analyses the adults subsample (15574 subjects 15 years and over, out of an estimated South African adult population of 34 million) of the first wave of the NIDS. The NIDS survey method is described in detail by Woolard et al. [20]. It was designed as a longitudinal panel survey of a nationally representative sample of households in South Africa. A two-stage cluster sample design was used to randomly select about 7300 households across 400 primary sampling units (areas), stratified by district council (a second level administrative division of South Africa’s territory in 53 areas). The first wave of the survey was conducted in 2008, and the target population was private households and residents in workers’ hostels, convents and monasteries, excluding other collective living quarters such as old age homes, hospitals, prisons and boarding schools. Trained fieldworkers were instructed to interview and collect anthropometric data on all available subjects belonging to the selected households. The household level response rate was 69% and the individual response rate within households was 93%. The NIDS study, the dataset of which is publicly available for research purposes [23], has been granted ethical approval by the Commerce Faculty Ethics Committee at the University of Cape Town.

### Measures

#### Sociodemographic variables

Age in years was treated as a continuous variable, and race self-defined by participants according to the historical “population group” categorization used in South Africa^a^. Education was measured in years of completed schooling, and individual monthly income was calculated as the summation of a wide array of sources, which is considered a more reliable method than the use of single questions [24]. Missing data in specific sources of income were imputed according to the procedure described by Argent [25].

#### Blood pressure and resting heart rate

Supine blood pressure and heart rate were measured twice by trained field workers in the left arm after a 5 minute rest period, using an automated blood pressure monitor (Omron M7 BP, multi-size cuff, factory calibrated). Measurements were retained if systolic blood pressure (SBP) was between 80 and 240 mm Hg, diastolic blood pressure (DBP) ≥ 30 mm Hg, and their difference was ≥ 15 mm Hg. Heart rate measurements were retained if ≥ 30 bpm and < 150 bpm.

#### Antihypertensive medication

Use of antihypertensives was assessed by asking subjects if they were currently taking medication for high blood pressure.

#### Bio-behavioural risk factors

BMI, smoking, alcohol use, physical exercise and resting heart rate were considered as possible mediators of the association between SES and blood pressure.

Duplicate measures of weight and height were recorded, with a third measure taken if their difference was greater than 0.5 Kg and 0.5 cm, respectively. Excluding measures with implausible values (height < 100 cm or > 200 cm, weight < 20 Kg or > 200 Kg), the average of the available readings was used to calculate BMI. Current smoking status, alcohol use and physical exercise were represented by ordinal variables, as shown in Table 1. Measurement procedures are detailed in the fieldwork manual of the NIDS [26].

**Table 1:** Descriptive statistics for the adult sub-sample of the National Income Dynamics Study

**Variable**	**N**	**Median/percentage**	**IQR/frequency**	**Range**
Men	15 574	40.2%	6 260	
Age	15 549			
15–24		30.4%	4 730	
25–34		18.9%	2 936	
35–44		16.8%	2 613	
45–54		14.0%	2 174	
55–64		10.0%	1 556	
65+		9.9%	1 540	
Race	15 574			
Black		78.5%	12 221	
Coloured		14.2%	1 215	
Asian		1.4%	224	
White		5.9%	914	
Individual income (ZAR)	15 276	600	[0 ; 1 200]	[0 ; 1 517 000]
Education	15 545			
None		14.0%	2 178	
Primary		16.7%	2 603	
Secondary		60.17%	9 353	
Tertiary		9.1%	1 411	
Average quantity of alcohol				
per drinking occasion	15 505			
Non drinker		75.8%	11 747	
1/2 standard drinks		7.2%	1 121	
3/4 standard drinks		6.7%	1 041	
5/6 standard drinks		4.8%	747	
7/8 standard drinks		2.3%	363	
9/12 standard drinks		1.7%	264	
13+		1.4%	222	
Ever smoked	15 505	25.6%	3 971	
Current smoking	15 227			
No		80.3%	12 230	
< 20 cigarettes/day		17.5%	2 658	
≥ 20 cigarettes/day		2.2%	339	
Physical exercise	15 471			
Never		70.1%	10 845	
< once a week		5.8%	900	
Once a week		5.6%	863	
Twice a week		6.1%	944	
≥ three times a week		12.4%	1 919	
SBP (mmHg)	13 852	121.5	[110 ; 137]	[80 ; 240]
DBP (mmHg)	13 836	79.5	[71 ; 89.5]	[31.5 ; 137]
HR (bpm)	14 025	75.5	[67 ; 84]	[32.5 ; 147]
BMI (kg/m ^2^)	13 858	24.4	[20.9 ; 29.7]	[7.1 ; 97.3]

N = number of nonmissing cases, IQR = Interquartile range. Values are unweighted.

### Statistical analysis

Sample characteristics were described as median and interquartile range for continuous variables and frequency for categorical measures.

**Figure 1:**
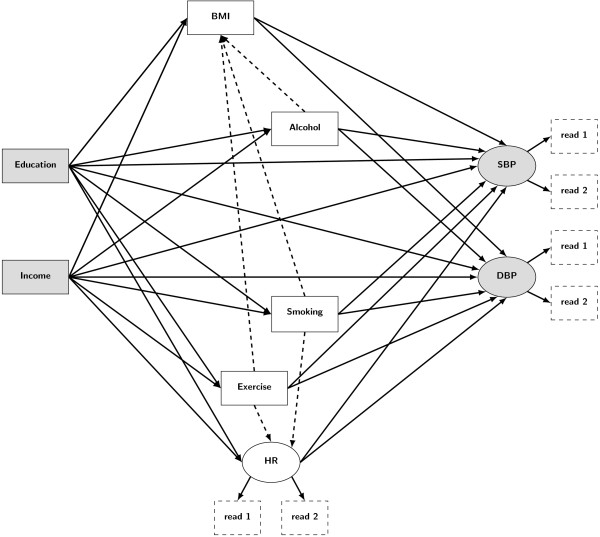
**Hypothesised causal pathways between education, income and blood pressure.** Squares and circles represent observed and latent variables, respectively. Arrows indicate hypothesised causal effects. Dashed squares indicate each of the multiple readings from which the values of the latent variables systolic blood pressure (SBP), diastolic blood pressure (DBP) and resting heart rate (HR) are inferred. Race, age and use of antihypertensive medication are omitted from the diagram, but taken into account as possible confounders in the model.

Based on epidemiological and biological evidence [13,27-31], we hypothesised that the variables considered in this study were causally linked as depicted in Figure 1. The model suggests a causal effect of education and income on SBP and DBP, partially mediated by BMI, heart rate, exercise frequency, alcohol use and smoking. It also assumes that BMI is affected by alcohol use, smoking and exercise, and that resting heart rate is influenced by smoking and exercise frequency.

Structural equation modelling was used to evaluate the extent to which the hypothesised causal structure was able to explain the observed associations between variables, and to estimate the magnitude of the postulated effects. To minimise the bias due to measurement error, blood pressure and heart rate were introduced as latent variables, with the observed multiple readings as indicators [32].

Estimated model coefficients were used to decompose total effects of SES on blood pressure (i.e. the change in blood pressure per unit increase in each of the SES indicators) into mediated and unexplained effects. Mediated effects (i.e. effects statistically explained by variations in BMI, smoking, alcohol use, physical exercise and heart rate) are represented in Figure 1 by indirect paths connecting SES indicators to blood pressure levels through the different factors. Unexplained effects (i.e. effects unrelated to variation in the considered mediators) are represented by direct paths connecting SES indicators to blood pressure.

Model coefficients were estimated adjusting for age, race and hypertensive medication. In view of previous evidence that relationships might differ by gender, models were fitted separately for women and men.

To relax the assumption of multivariate normality underlying the estimation of standard errors with the usual formulae – rarely satisfied in multiple mediators models – 95% confidence intervals for mediated and unexplained effects were bootstrapped, and effects considered statistically significant at probability level *α*=0.05 when the confidence interval excluded the null value.

Analyses were carried out using Stata®; 12 and Mplus®; 6 [33,34], taking into account the complex sampling scheme of the NIDS.

Further details on modelling assumption and estimation procedure are reported in Additional file 1.

## Results

Unweighted sample characteristics are described in Table 1. The great majority of participants were Black and Coloured. Whites were under-represented relative to the South African population, owing to their low response rate in the NIDS [20]. Using SBP ≥ 140 mm Hg and/or DBP ≥ 90 mm Hg as cut-offs [35], 28.4% of male participants and 30.6% of female participants would be classified as hypertensive. Current use of antihypertensives was reported by 7.4% of men and 16% of women.

Table 2 shows the estimated average blood pressure and hypertension prevalence in the South African adult population. The comparison of these estimates with those from the 1998 South African Demographic and Health Survey [36] (not shown), suggests that in the last 10 years the prevalence of hypertension among South African adults has increased considerably (proportionately by 22% in men and 28% in women) [37].

**Table 2:** Average blood pressure and prevalence of hypertension in the South African adult population

	**Women**		**Men**	
**Variable**	**Estimate**	**95% CI**	**Estimate**	**95% CI**
SBP (mmHg)	122.8	[122.0 ; 123.7]	125.7	[124.8 ; 126.7]
DBP (mmHg)	80.6	[80.0 ; 81.3]	78.9	[78.2 ; 79.6]
Hypertension prevalence (%)	33.5	[31.5 ; 35.4]	28.0	[26.0 ; 30.0]
Subjects on antihypertensive medication (%)	13.3	[12.0 ; 14.7]	5.8	[4.9 ; 6.7]

Estimates take into account the complex sample design of the NIDS. Subjects on medication are considered hypertensive, regardless of their blood pressure readings.

### Association of education and income with blood pressure

The structural models showed an excellent fit with the data (see Table 3), supporting our hypothesis that the causal structure in Figure 1 is a plausible explanation of the observed associations between variables. The estimated values of the model coefficients (and corresponding confidence intervals) are listed in the additional material and qualitatively summarised in Table 4.

**Table 3:** Fit indices for the structural models

**Model**	***χ***^**2**^	***χ***^**2**^**/*d*** ***f***	**RMSEA**	**CFI**	**TLI**	**WRMR**
Men	49.04	1.17	0.005	0.999	0.996	0.269
	*df*=42, *p*=0.211		90% CI=[0.000 ; 0.011], *p*-*close*>0.999			
Women	56.32	1.34	0.006	0.998	0.994	0.330
	*df*=42, *p*=0.070		90% CI=[0.000 ; 0.010], *p*-*close*>0.999			

*χ*^2^= Chi-squared test of model fit, RMSEA = Root Mean Square Error of Approximation, *p* - *close* = probability of RMSEA < 0.05, CFI = Comparative Fit Index, TLI = Tucker Lewis Index, WRMR = Weighted Root Mean Square Residual.

**Table 4:** **Sign and statistical significance of the estimated path coefficients for the model in Figure**1

	**Dependent variable**													
	**Women**							**Men**						
**Independent variable**	**SBP**	**DBP**	**BMI**	**Alcohol**	**Smoking**	**Exercise**	**HR**	**SBP**	**DBP**	**BMI**	**Alcohol**	**Smoking**	**Exercise**	**HR**
Education	▾	▾	▴	▵	▾	▴	▾	▿	▵	▴	▴	▾	▴	▿
Income	▾	▿	▴	▴	▵	▿	▿	▵	▴	▴	▴	▴	▿	▿
BMI	▴	▴						▴	▴					
Alcohol	▵	▴	▵					▵	▵	▵				
Smoking	▿	▿	▾				▴	▿	▿	▾				▴
Exercise	▿	▵	▾				▿	▵	▵	▿				▾
HR	▿	▴						▵	▴					

*Note:* ▴ / ▵ = An increase in the value of the independent variable predicts an increase in the value of the dependent variable; ▾ / ▿ = An increase in the value of the independent variable predicts a decrease in the value of the dependent variable; Filled/hollow symbols = Statistically significant/non significant coefficients (*α*=5*%*).

The estimated total effects indicated that, among women, each year of education was associated with 0.29 mm Hg *drop* in SBP and 0.12 mm Hg *drop* in DBP. A doubling of the monthly income was similarly related to a *decrease* of SBP by 0.15 mm Hg, while the association of income with DBP was trivial in magnitude and not statistically significant.

In men, by contrast, an increase in both education and income was associated with an *increase* in blood pressure levels, but only the relationships with DBP were statistically significant, indicating an increase of 0.11 mm Hg per year of education and 0.12 mm Hg for each income doubling.

Overall then, the findings show an inconsistent relationships between SES indicators and blood pressure across gender. Education and income have positive (or null) associations with blood pressure levels among men (*harmful* effect of increased SES on blood pressure), but inverse (or null) associations among women (*protective* effect). The relative size of the coefficients and the width of their confidence intervals suggest also that SES is more strongly associated with DBP than SBP in men, while the opposite holds for women.

### Mediation

**Figure 2:**
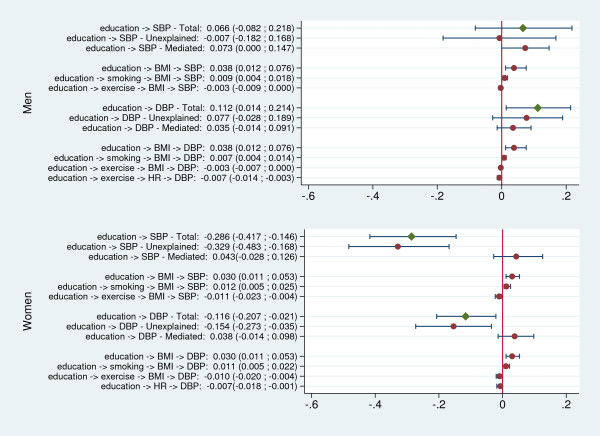
**Mediated, unexplained and total effects of education on blood pressure, and statistically significant specific pathways.** Values represent the average increase in blood pressure (in mm Hg) per year of education.

**Figure 3:**
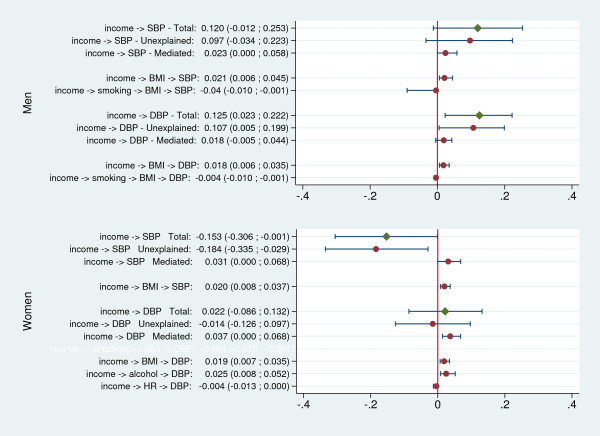
**Mediated, unexplained and total effects of (log) income on blood pressure, and statistically significant specific pathways.** Values represent the average increase in blood pressure (in mm Hg) when the income doubles.

Figures 2 and 3 summarize the results of the mediation analysis.

Among men, BMI was, by far, the strongest mediator of the harmful effect of increasing SES on blood pressure. All statistically significant indirect paths included BMI, with the only exception being the one connecting education to DBP though exercise and resting heart rate. Overall, the sum of the effects mediated by BMI in men accounted for a 0.042 mm Hg increase in SBP per year of education (95% CI: 0.014 to 0.084) and a 0.019 mm Hg increase per income doubling (95% CI: 0.005 to 0.044). The corresponding values for DBP were 0.035 (95% CI: 0.014 to 0.063) and 0.015 (95% CI: 0.005 to 0.034).

Smoking, exercise frequency and heart rate were also involved in significant relationships in men, some of them (involving exercise and smoking through BMI and HR) representing protective effects, opposite to the total effect. However, their overall role was modest compared to that of BMI. The only statistically significant direct path (representative of an unexplained harmful effect) connected income to DBP.

As among men, BMI in women mediated statistically significant harmful effects, despite the overall protective role of higher SES on blood pressure. Effects mediated by BMI accounted for a 0.028 mm Hg increase in SBP per year of education (95% CI: 0.014 to 0.056) and a 0.019 mm Hg per income doubling (95% CI: 0.010 to 0.034) as well as a 0.028 mm Hg increase in DBP per year of education (95% CI: 0.014 to 0.056), and a 0.019 mm Hg per income doubling (95% CI: 0.005 to 0.034).

Independently of its effect on BMI, alcohol use in women accounted for a 0.024 mm Hg increase in DBP per income doubling (95% CI: 0.010 to 0.053). The mediating role of the remaining risk factors was similar to that of men, both in magnitude and direction. Except for the effect of income on DBP, all direct paths in women were statistically significant and accounted for a sizable share of the overall association between SES and blood pressure, which remained therefore largely unexplained by the mediators hypothesised in our model.

## Discussion

Relationships between SES and blood pressure with gender specific patterns consistent with our results (i.e. a protective effect of SES among women and a harmful effect among men) have been previously found in South Africa [5] and other middle-income countries [38-40]. These findings are in partial contrast with those in high income countries, where an inverse gradient SESblood pressure is commonly found in both genders, even though often stronger and more consistent across SES indicators in women than in men [2]. In our study, total mediated effects (i.e. the sum of the effects through all indirect pathways) were similar in direction and magnitude across genders, and therefore the observed discrepancies cannot be explained by gender differences in the distribution of the hypothesised mediators. It is conversely the presence – in women but not in men – of sizable unexplained protective effects outmatching the overall mediated effects (harmful in both genders) which makes the difference and suggests that the inverse effect of SES on blood pressure observed in women is mediated mainly by factors not included in our analysis.

Increased awareness of hypertension, accessibility of and adherence to medical treatment, less chronic stress, and, recently, more favourable neighbourhood characteristics, have been indicated in the literature as possible mediators of a protective effect of higher SES on raised blood pressure, and may contribute to the share of effect among women which is unexplained in our model. The reason why a protective effect of the same magnitude is not observed in men might be related to the lower levels of awareness and control consistently observed in men than in women, and to the lower sensitivity to the adverse effect of unfavourable neighbourhood characteristics on blood pressure that recent studies suggest [2,18,41-44].

Salt intake, whose causal relationship with blood pressure levels is supported by results of experimental studies in South Africa [41], has also been proposed as a possible mediator [2]. Nevertheless, results from two large scale surveys of the South African population suggests a positive association between SES and salt intake, making this factor a candidate mediator for the unexplained portion of the harmful effect of income on DBP in men, but not for the protective effects of SES in women [42,45].

Beyond differences in relative magnitude and statistical significance, the overall patterns of association of physical exercise, alcohol use, smoking and heart rate with both SES and blood pressure are consistent with those found in high income countries [18,19]. However, in contrast to those studies but coherently with other studies in sSA [6,46], in our population BMI rises with increasing SES. It therefore mediates a harmful effect of increasing SES on blood pressure, accounting for a sizable proportion of the association in men, and contributing to reducing the overall protective effect found in women. These contrasting results may be partly explained by considering that the distribution of income in South Africa is extremely unequal, and, despite the mean income per capita being relatively high, a substantial proportion of the population lives near or below the poverty line [47]. It is likely that among people in this setting the increased knowledge of health risk and greater motivation to control weight associated with increasing SES – which have been argued as an explanation of the inverse SES/BMI relationship in high income countries – play a less significant role than the greater access to energy dense processed food among those with higher SES.

A separate analysis for the 5% of the total sample with the highest income offers some support for this hypothesis. In that subsample the associations between SES indicators and BMI become inverse also in men, albeit not statistically significant owing to the small sample size (see Additional file 1).

Finally, we found that socioeconomic variables affect DBP more strongly than SBP among men and vice versa among women. This different responsiveness may explain some incongruences between results of studies using only systolic blood pressure as the outcome variable and studies analysing hypertension prevalence (defined in terms of both SBP and DBP). This heterogeneity is not accounted for by the modest differences in the association between mediators and SBP or DBP, and calls for consideration of other variables. Among those, stress and dietary patterns (e.g. vegetable consumption) have been shown to be selectively associated with SBP and DBP, thus representing suitable targets for further mediation studies [48,49].

Strengths of the present study include the use of a large sample and an analytical approach allowing for simultaneous testing of multiple mediation pathways (avoiding the potential bias arising from neglecting the correlation between mediators) and for the explicit consideration of measurement error in physiological variables. Moreover, this study is the first, to our knowledge, to perform mediation analysis in a large sample modelling simultaneously for both SBP and DBP.

The major limitations of this study are the intrinsic lack of temporal information in our cross-sectional dataset – which limits the interpretation of the temporal sequence of the relationships – and the possibility that important unmeasured confounding variables (e.g. undernutrition in infancy which could be associated with income and is a known risk factor for high blood pressure later in life) [50] have introduced bias into the study results.

Low reliability of self-report data, including those on physical exercise, alcohol and tobacco use is a well known problem in population-based surveys, which usually results in observed associations biased towards the null [51]. More precise measurements are therefore likely to strengthen the result of our analysis rather than invalidate them.

Other than for age – a strong predictor of blood pressure which is associated with many of the variables in our model – our analyses were adjusted for racial group and antihypertensive medication. Despite the fact that statistical control for race and medication is common in the literature (see for example [6,18,19]), we cannot exclude the possibility that these variables act instead as effect modifier and mediator respectively.

Besides income and education, racial group assignment may indirectly capture differences in household wealth, genetic ancestry, social stress (e.g. migration, discrimination) and dietary intake, possible confounders of the association between income and blood pressure not otherwise captured in our analyses [52-57]. These considerations justify its introduction as a confounder in the models. However, the legacy and persistence of separate educational systems for the different racially defined groups [58] and the likely differential economic value of a given educational level [59] make it plausible that the value of education as a measure of SES differs by racial group. Race would thus be an effect modifier, as well as a confounder, of the hypothesised causal relationship between years of schooling and blood pressure [1].

Similarly, we cannot rule out that being on medication for high blood pressure (which is positively correlated with income and education in our sample, after adjustment for age and gender) lies on a causal pathway between SES and blood pressure (higher SES → increased access to health care → increased use of antihypertensive drugs → lower blood pressure). In this case, adjustment for a mediator would bias the observed association between SES and blood pressure towards the null.

However, restricting our analyses to the Black subsample or omitting adjustment for antihypertensive medication did not produce appreciable changes in the overall pattern of association between variables. All model coefficients maintained the same sign with negligible to moderate changes in magnitude (see Additional file 1), thus supporting the robustness of the results of this study to incorrect specification of the role of these variables.

Finally, the specific characteristics of the highly uneven socio-economic development of South Africa make it necessary to exercise a degree of caution in generalizing the results of this study to other areas of sSA or other developing countries. The findings need replication in other settings with rapid but complex epidemiologic transitions.

## Conclusion

The findings of this study strengthen the case that SES is associated with hypertension and that physical exercise, alcohol use, smoking, resting heart rate and BMI play a role in explaining socioeconomic inequalities in blood pressure. However, in contrast to most studies in high income populations, our results suggest that in settings such as South Africa, characterised by rapid and complex epidemiological transition (1) effects of SES on blood pressure may vary by gender; and (2) factors other than those listed above may have a greater role in mediating the association in women.

The worsening in blood pressure with upward mobility observed in men can be viewed as a significant public health cost of socio-economic development, while understanding the causes of the opposite effect in women may inform action to reduce the growing cardiovascular health burden in developing countries.

## Endnote

^a^ Under apartheid, South Africans were categorised into one of four socially defined groups: Asian (or Indian), Black (or African), Coloured (wide grouping of people of mixed ancestry) and White (or European). Race in this sense is closely and enduringly correlated with socioeconomic status in South Africa.

## Competing interests

The authors declare that they have no competing interests.

## Authors’ contributions

AC performed the analysis and drafted the manuscript. RE conceived the study and contributed to the drafting of the manuscript. Both authors read and approved the final manuscript.

## Pre-publication history

The pre-publication history for this paper can be accessed here:

http://www.biomedcentral.com/1471-2458/14/414/prepub

## Supplementary Material

Additional file 1 **Statistical analyses and further results.** Methodological details on statistical analyses. Further results. Click here for file
