# Supplementary material for: Analysing the socioeconomic determinants of hypertension in South Africa: a structural equation modelling approach
Source: BMC Public Health. 2014 May 1;14:414. doi: 10.1186/1471-2458-14-414 (PMC4021547; doi:10.1186/1471-2458-14-414)
Supplement: Additional file 1 — Statistical analyses and further results. Methodological details on statistical analyses. Further results. [file 1471-2458-14-414-S1.pdf]

ONLINE SUPPLEMENT

Analysing the socioeconomic determinants of hypertension in South Africa: a structural equation modelling approach

Annibale Cois, Rodney Ehrlich

School of Public Health and Family Medicine
University of Cape Town, Anzio Road, Observatory 7925
Cape Town, South Africa

Table of Contents:

Additional notes on measures and data transformation 2
Additional notes on statistical analyses 2
Mplus code 5
Model fit indices 6
Coefficients estimates 6
Association between SES and blood pressure among subject with high income 6
Sensitivity analysis 6
References 7
Tables 8

Additional notes on measures and data transformation

Income: The distribution of the variable income was extremely skewed in our sample, 90% of subjects earning less than $ZAR\,3300/month$, while the remaining 10% had a monthly income between $ZAR\,3300$ and 1.5 million. Even if income is introduced in the models only as a predictor, and therefore no assumptions need to be made about its distribution, we adopted the common practice in econometrics of log-transforming this variable. This was based on the reasonable assumption that the impact on the subject's lives of a given income increment decreases as income increases, and does not stay constant as implied by the untransformed variable. As a secondary benefit, log-transformation reduces the dependency of the estimated regression coefficients on extreme values, avoiding an excessive influence by the (few) subjects in the sample with very large income. A natural logarithm transformation is used in the analyses, but the results in the article are rescaled so they refer to the effect of doubling the income.

Age: The finding of the great majority of studies in literature show that in most population — with a few exceptions — the average values of both systolic and diastolic blood pressure rise during childhood and adulthood; thereafter systolic pressure maintains the trend until the eighth or ninth decade, while diastolic pressure tends to decline slightly after the age of 55/60 years.[1] Because of this non-linear relationship, which is confirmed in our sample (see Figure 1 and also the relative size of the regression coefficients for age1 and age2 on blood pressure in the structural model), age was introduced in our models as a linear spline with a single knot corresponding to 55 years, in order to reduce residual confounding due to improper adjustment.

Additional notes on statistical analyses

Sampling weights: The analyses were adjusted for survey design effect, taking into account the clustering, stratification and sampling weights. Untrimmed post-stratification sampling weights (version 4.1) were utilised for the adjustment.[2] In the National Income Dynamics Study (NIDS) survey — owing to the adjustment for the largely unequal response rate among population groups, geographical regions and age classes and the calibration procedure — sampling weights show a very large variation, ranging from 0.57 to 29 545, and this is known to produce excessively large confidence intervals in the estimates. However, we accepted this likely reduction in precision and we avoided utilising trimmed weights (also provided in the dataset) which are an acknowledged source of bias in point estimates.[3]

Estimation of population averages of blood pressure and prevalence of hypertension: Population averages of blood pressure and prevalence of hypertension were estimated from the sample using Stata® ver. 12.[4] Confidence intervals were adjusted for the sampling scheme of the NIDS using the Taylor linearization method.

Figure 1: Average systolic (SBP) and diastolic (DBP) blood pressure vs. age in the sample†

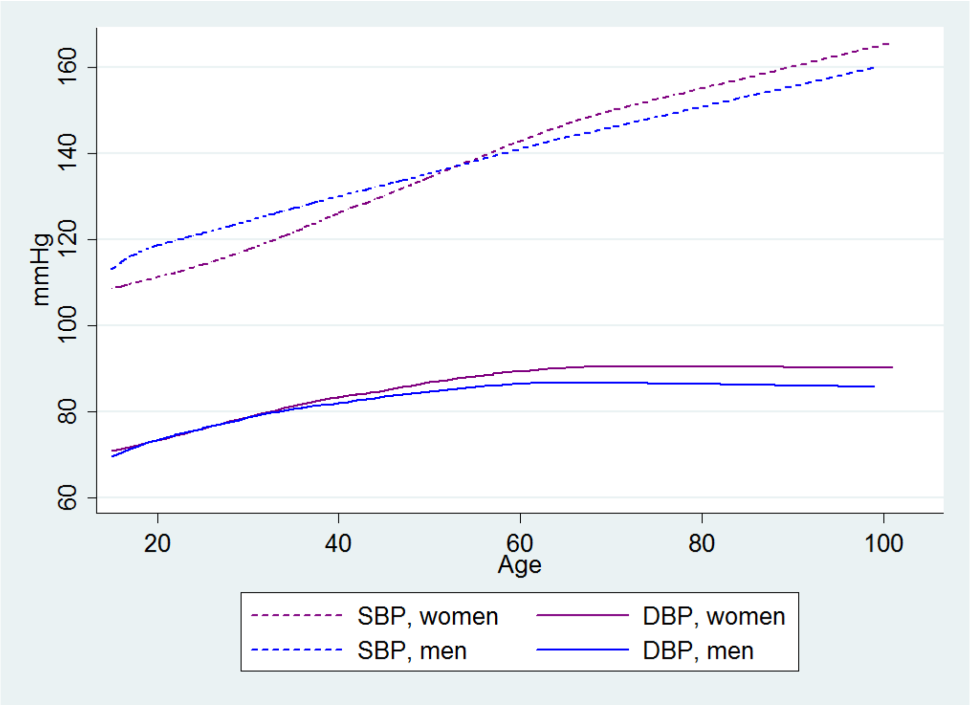

† Smoothed curves. Locally weighted regression (Stata® *lowess* command, default bandwidth)

Estimation of structural models: We used Mplus® ver. 6.12 to estimate the structural path models.[5]

Because of the presence of three categorical mediators (*exercise*, *alcohol* and *smoking*), we used the weighted least-squares with mean and variance adjustment (WLSMV) estimation procedure for which there seems to be growing consensus in literature. Despite its lower efficiency and greater computational requirements, the WLSMV estimator offers substantial advantages over the traditional maximum likelihood (ML) estimator when ordinal variables with less than 5 categories and/or with a highly non-normal distribution are introduced in the model. This is the case in our dataset, in which *smoking* is coded with only three categories and *exercise* and *alcohol* — despite having 5 and 7 categories, respectively — show a left-skewed distribution and large values for kurtosis (especially among women). It has been shown that, in these condition, ML estimator tend to underestimate regression coefficients, and overestimate the values for the χ^2 statistic, leading to an increased risk of rejecting a model which fits the data adequately.[6] The values of the χ^2 statistic reported in the article were adjusted to take into account this bias, according to the procedure described by Muthén.[7]

With WLSMV a categorical (ordinal) variable is considered as the expression of an underlying continuous latent response variable categorized using a set of thresholds (estimated with a *probit* model) and it is the latent variable which is introduced in the structural model. As a consequence, the estimated regression coefficients in relationships involving categorical variables represents linear regression coefficients for the *continuous latent*

response and not for the original variable.[6]

It is worth noticing that, when the coefficients of a path connecting a predictor (education or income) to an outcome (systolic of diastolic blood pressure) are multiplied to obtain the overall effect, the metric of the result depends only on the scale of the predictor and the outcome, and, therefore, can be interpreted as a linear regression coefficient.[8]

Missing data were managed with a modified version of pairwise deletion as described in Asparouhov and Muthén.[9]

Latent Variables: To minimise the bias due to measurement error, blood pressure and heart rate were introduced as latent variables, with the observed multiple readings as indicators. Latent variables are not directly observed but rather analytically inferred from other variables directly measured (indicators). They are used in structural equation modelling either to represent abstract concepts (like mental states) or as in our case aspects of physical reality which could in principle be measured but may not be for practical reasons, including measurement error. Using latent variables allows the estimation and removal of the measurement error associated with the observed variables. In the case of blood pressure measurement, this procedure have been shown, under relatively broad assumptions, to be more effective than the common practice of averaging multiple readings.[10]

Magnitude of mediated and unexplained effects: The magnitude of mediated effects, i.e. the amount by which blood pressure is expected to increase (or decrease) per a unit change in education or (log)income as a result of the variation of the involved factors, was calculated as the product of the regression coefficients in the considered paths.[8] The magnitude of unexplained effects was estimated by the coefficients of the direct paths connecting SES indicators to blood pressure, and total effects were calculated as the sum of the unexplained and all mediated effects.

Rescaling: Due to the large differences in the variances of the continuous variables in their original scales (ill-scaled covariance matrix) systolic blood pressure (SBP), diastolic blood pressure (DBP) and heart rate (HR) were rescaled to reduce convergence problems in the estimation algorithm.[11] Model coefficients were reported in the original scale in the article.

Mplus code

```
Title:
  MEDIATION MODEL

Data:
  File is ********* ;

Variable:
 Names are
     age1 age2                      ! linear spline for age
     gender                         ! gender
     bla asi col whi                ! dummies for racial groups
     htnmed                         ! antihypertensive medication
     sys1 sys2                      ! duplicate readings of systolic blood pressure
     dia1 dia2                      ! duplicate readings of diastolic blood pressure
     pul1 pul2                      ! duplicate readings of resting heart rate
     l_inc                          ! natural logarithm of income
     edu                            ! years of education
     alcq                           ! alcohol use
     exerc                          ! exercise frequency
     smokcat                        ! smoking
     bmi                            ! body mass index
     psu stratum sweight;           ! sampling design variables

  Missing are all (-9999) ;
  stratification is stratum;
  cluster is psu;
  weight is sweight;
  subpopulation IS gender EQ 1;     ! gender=1 for men, gender=0 for women

  usevariables ARE age1 age2 htnmed col asi whi exerc smokcat alcq l_inc edu
                 pul1a pul2a dia1a dia2a sys1a sys2a bmia;
  categorical ARE  exerc alcq smokcat;

 Define:                             ! rescaling
  pul1a=pul1/5;
  pul2a=pul2/5;
  sys1a=sys1/7;
  sys2a=sys2/7;
  dia1a=dia1/7;
  dia2a=dia2/7;
  bmia=bmi/5;

Analysis:
  type=complex;
  reps=BOOTSTRAP;
  bootstrap=2000;

Model:
 SBP BY sys1a sys2a;
 DBP BY dia1a dia2a;
 HR by pul1a pul2a;
 SBP ON age1 age2 htnmed col asi whi exerc smokcat alcq bmia HR edu l_inc;
 DBP ON age1 age2 htnmed col asi whi exerc smokcat alcq bmia HR edu l_inc;
 bmia ON age1 age2 htnmed col asi whi alcq smokcat exerc edu l_inc;
 HR ON age1 age2 htnmed col asi whi smokcat exerc edu l_inc;
 exerc ON age1 age2 htnmed col asi whi edu l_inc;
 smokcat ON age1 age2 htnmed col asi whi edu l_inc;
 alcq ON age1 age2 htnmed col asi whi edu l_inc;
 sys1a WITH dia1a;
 sys2a WITH dia2a;
 SBP WITH DBP;

 smokcat WITH alcq;
 HR WITH alcq;                      ! only males
 HR WITH bmia;                      ! only males
 smokcat WITH exerc;                ! only females
 exerc WITH alcq;                   ! only females

Output:
RESIDUAL;
CINTERVAL (BCBOOTSTRAP);
```

Note: Non-causal correlations (i.e. spurious associations not explained by the variables included in the model) were allowed between each pair of blood pressure measurements and between the latent variables representing systolic and diastolic blood pressure. Allowing for these correlations to be different from 0 means accepting the plausible hypotheses that (1) factors related to the specific conditions of the measurement (e.g. cuff positioning, procedure used by the fieldworker) affect the measured values of systolic and diastolic blood pressure in the same reading, creating a correlation which is not completely explained by the "true" values of the blood pressure; and (2) that systolic and diastolic blood pressure are affected by factors not considered in our analysis (e.g. genetic characteristics of the individuals).

Moreover, according to the convincing suggestion of Preacher and Hayes,[12] we did not constrain the residual variances of the mediators (more precisely, the residual variances of the latent variables representing the ordinal mediators) to be uncorrelated in principle, and we introduced non-causal paths (WITH statements in the model above) between mediators for which no causal relationship was hypothesised, when beneficial for model fit.

Model fit indices

The structural models showed an excellent fit with the data (see indices Table 3 in the Article). In particular the non significant *p-values* associated with the χ^2 statistics supported our hypothesis that the causal structure in Figure 1 in the article is a plausible explanation of the observed associations between variables.[13]

Coefficients estimates

Table 1 shows the unstandardised coefficients for the hypothesised causal paths — estimated separately for men and women — and the corresponding 95% confidence intervals.

Association between SES and blood pressure among subject with high income

In the 5% of the total sample with the highest income, linear regression coefficients between SES indicators and BMI, adjusted for age, race and gender) were -0.04 (95%CI: -0.17 to 0.09) for education and -0.34 (95%CI: -0.85 to 0.17) for (log) income.

Sensitivity analysis

Table 2 compares the coefficients of the fully adjusted model to the coefficients estimates restricting the analyses to the Black subsample and omitting adjustment for antihypertensive medication.

References

1 Bazzano LA, He J, Whelton PK: Blood pressure in westernized and isolated populations. In *Comprehensive hypertension*. Edited by Lip GYH, Hall JE, Philadelphia: Mosby, Esevier 2007:21–30.

2 Wittemberg M: *Weights: Report on NIDS Wave 1. Technical Paper no. 2.* Cape Town: Southern Africa Labour and Development Research Unit 2009.

3 Potter FJ: *Methods for extreme weights in sample surveys. PhD Thesys.* Chapel Hill: Department of Biostatistics, University of North CaTo1ina 1990.

4 StataCorp: *Stata Statistical Software: Release 12.* College Station, TX: StataCorp LP 2011.

5 Muthén L, Muthén B: *Mplus Users Guide*. Los Angeles, CA: Muthén & Muthén, 6th edition 2011.

6 Finney SJ, DiStefano C: Non-normal and categorical data in structural equation modeling. In *Structural Equation Modeling: A Second Course*. Edited by Hancock GR, Mueller RO, Greenwich: Information Age Publishing 2006:269–312.

7 Muthén B, Asparouhov T: Simple Second Order Chi-Square Correction. MPlus Technical Appendices.

8 Preacher KJ, Kelley K: Effect size measures for mediation models: Quantitative strategies for communicating indirect effects. *Psychol Methods* 2011, **16**(2):93–115.

9 Asparouhov T, Muthén B: Weighted least squares estimation with missing data. Technical Report.

10 Batista-Foguet JM, Coenders G, Ferragud MA: Using structural equation models to evaluate the magnitude of measurement error in blood pressure. *Stat Med* 2001, **20**(15):2351–2368.

11 Kline RB: *Principles and Practice of Structural Equation Modeling.* New York: Guilford Press, 3rd edition 2011.

12 Preacher KJ, Hayes AF: Asymptotic and resampling strategies for assessing and comparing indirect effects in multiple mediator models. *Behav Res Meth* 2008, **40**(3):879–891.

13 Hooper D, Coughlan J, Mullen MR: Structural equation modelling: guidelines for determining model fit. *EJBRM* 2008, **6**:53–60.

Tables

Table 1: Coefficients estimates

	Women		Men	
Path	Coeff	95% CI	Coeff	95% CI
SBP → SYS1A	1.000	1.000 ; 1.000	1.000	1.000 ; 1.000
SBP → SYS2A	0.991	0.967 ; 1.033	1.048	1.007 ; 1.120
DBP → DIA1A	1.000	1.000 ; 1.000	1.000	1.000 ; 1.000
DBP → DIA2A	0.980	0.950 ; 1.028	1.017	0.968 ; 1.075
HR → PUL1A	1.000	1.000 ; 1.000	1.000	1.000 ; 1.000
HR → SBP	-0.014	-0.058 ; 0.027	0.016	-0.031 ; 0.073
HR → DBP	0.035	0.007 ; 0.064	0.074	0.043 ; 0.107
AGE1 → SBP	0.091	0.079 ; 0.102	0.068	0.056 ; 0.079
AGE2 → SBP	0.076	0.044 ; 0.108	0.058	0.027 ; 0.089
HTNMED → SBP	1.327	0.958 ; 1.671	0.631	-0.078 ; 1.340
COL → SBP	0.445	0.002 ; 0.968	0.583	0.107 ; 0.978
ASI → SBP	-0.036	-0.792 ; 1.027	-0.072	-0.813 ; 1.507
WHI → SBP	-0.140	-0.780 ; 0.466	-0.270	-0.706 ; 0.195
EXERC → SBP	-0.032	-0.157 ; 0.094	0.046	-0.072 ; 0.166
SMOKCAT → SBP	-0.071	-0.324 ; 0.176	-0.032	-0.186 ; 0.118
ALCQ → SBP	0.066	-0.110 ; 0.245	0.072	-0.073 ; 0.213
BMIA → SBP	0.188	0.121 ; 0.260	0.179	0.069 ; 0.296
EDU → SBP	-0.047	-0.069 ; -0.024	-0.001	-0.026 ; 0.024
L_INC → SBP	-0.038	-0.069 ; -0.006	0.020	-0.007 ; 0.046
AGE1 → DBP	0.054	0.046 ; 0.061	0.051	0.043 ; 0.058
AGE2 → DBP	-0.008	-0.025 ; 0.010	-0.013	-0.032 ; 0.005
HTNMED → DBP	0.698	0.444 ; 0.926	0.259	-0.139 ; 0.642
COL → DBP	0.251	-0.098 ; 0.657	0.344	0.076 ; 0.623
ASI → DBP	-0.060	-0.824 ; 0.543	-0.083	-0.461 ; 0.864
WHI → DBP	-0.227	-0.618 ; 0.182	-0.163	-0.477 ; 0.153
EXERC → DBP	0.026	-0.070 ; 0.112	0.008	-0.071 ; 0.089
SMOKCAT → DBP	-0.011	-0.185 ; 0.169	-0.033	-0.146 ; 0.072
ALCQ → DBP	0.159	0.045 ; 0.271	0.058	-0.034 ; 0.146
BMIA → DBP	0.179	0.127 ; 0.232	0.149	0.078 ; 0.223
EDU → DBP	-0.022	-0.039 ; -0.005	0.011	-0.004 ; 0.027
L_INC → DBP	-0.003	-0.026 ; 0.020	0.022	0.001 ; 0.041
AGE1 → HR	-0.018	-0.027 ; -0.009	0.013	0.004 ; 0.023
AGE2 → HR	-0.009	-0.029 ; 0.010	-0.017	-0.040 ; 0.006
HTNMED → HR	0.379	-0.036 ; 0.713	0.220	-0.161 ; 0.550
COL → HR	-0.020	-0.424 ; 0.416	0.000	-0.391 ; 0.464
ASI → HR	0.508	-0.545 ; 1.707	0.525	-0.547 ; 1.297
WHI → HR	-0.390	-0.935 ; 0.155	0.139	-0.423 ; 0.698
SMOKCAT → HR	0.220	0.062 ; 0.362	0.174	0.061 ; 0.280
EXERC → HR	-0.016	-0.123 ; 0.082	-0.253	-0.385 ; -0.126
EDU → HR	-0.030	-0.050 ; -0.009	-0.013	-0.039 ; 0.013
L_INC → HR	-0.025	-0.051 ; 0.003	-0.003	-0.031 ; 0.024
AGE1 → BMIA	0.035	0.029 ; 0.040	0.025	0.021 ; 0.029
AGE2 → BMIA	-0.038	-0.050 ; -0.027	-0.026	-0.037 ; -0.014
HTNMED → BMIA	0.698	0.529 ; 0.894	0.521	0.293 ; 0.733
COL → BMIA	0.332	0.052 ; 0.621	0.159	-0.067 ; 0.364
ASI → BMIA	-0.181	-0.835 ; 0.167	0.181	-0.090 ; 0.602
WHI → BMIA	0.129	-0.200 ; 0.486	0.364	0.132 ; 0.564
ALCQ → BMIA	0.001	-0.113 ; 0.111	0.020	-0.046 ; 0.079
SMOKCAT → BMIA	-0.233	-0.371 ; -0.095	-0.185	-0.280 ;-0.100
EXERC → BMIA	-0.135	-0.233 ; -0.046	-0.045	-0.109 ; 0.011
EDU → BMIA	0.022	0.008 ; 0.034	0.030	0.019 ; 0.042
L_INC → BMIA	0.022	0.008 ; 0.036	0.024	0.011 ; 0.038
AGE1 → EXERC	-0.008	-0.014 ; -0.003	-0.027	-0.032 ;-0.023
AGE2 → EXERC	-0.009	-0.020 ; 0.002	0.011	-0.002 ; 0.023
HTNMED → EXERC	0.149	-0.031 ; 0.311	0.084	-0.166 ; 0.310
COL → EXERC	0.393	0.218 ; 0.571	0.084	-0.132 ; 0.312
ASI → EXERC	0.922	0.591 ; 1.431	0.099	-0.441 ; 0.371
WHI → EXERC	1.035	0.861 ; 1.208	0.392	0.166 ; 0.598
EDU → EXERC	0.061	0.051 ; 0.070	0.055	0.042 ; 0.067
L_INC → EXERC	-0.002	-0.018 ; 0.014	-0.004	-0.018 ; 0.008
AGE1 → SMOKCAT	0.006	-0.002 ; 0.013	0.017	0.011 ; 0.022
AGE2 → SMOKCAT	-0.035	-0.053 ; -0.019	-0.064	-0.079 ; -0.050
HTNMED → SMOKCAT	-0.156	-0.369 ; 0.065	-0.360	-0.588 ; -0.133

Table 1: Coefficients estimates (continue)

	Women		Men	
Path	Coeff	95% CI	Coeff	95% CI
COL → SMOKCAT	1.519	1.327 ; 1.665	0.501	0.357 ; 0.633
ASI → SMOKCAT	0.353	-2.477 ;0.977	0.458	-0.036 ; 0.736
WHI → SMOKCAT	1.746	1.477 ; 2.016	0.789	0.546 ; 1.032
EDU → SMOKCAT	-0.039	-0.055 ;-0.022	-0.038	-0.050 ;-0.026
L_INC → SMOKCAT	0.004	-0.019 ; 0.029	0.024	0.007 ; 0.040
AGE1 → ALCQ	-0.005	-0.010 ; 0.001	0.009	0.005 ; 0.014
AGE2 → ALCQ	-0.008	-0.018 ; 0.001	-0.022	-0.033 ; -0.013
HTNMED → ALCQ	-0.078	-0.218 ; 0.059	0.009	-0.129 ; 0.135
COL → ALCQ	0.695	0.528 ; 0.849	0.190	0.012 ; 0.359
ASI → ALCQ	0.456	-0.161 ; 1.287	0.025	-0.432 ; 0.348
WHI → ALCQ	1.020	0.804 ; 1.221	0.112	-0.073 ; 0.302
EDU → ALCQ	0.006	-0.007 ; 0.019	0.011	0.000 ; 0.021
L_INC → ALCQ	0.033	0.019 ; 0.048	0.029	0.012 ; 0.046

Table 2: Comparison of the coefficients in the fully adjusted model (Full) with those estimated with restriction to the Black subsample (Res) and with no adjustment for antihypertensive medication (Med)

	Women			Men		
Path	Full	Res	Med	Full	Res	Med
HR → SBP	-0.01	-0.01	-0.01	0.02	0.02	0.02
HR → DBP	**0.03**	**0.04**	**0.04**	**0.07**	**0.07**	**0.07**
EXERC → SBP	-0.03	-0.10	-0.01	0.05	0.06	0.04
SMOKCAT → SBP	-0.08	-0.07	-0.10	-0.03	-0.04	-0.06
ALCQ → SBP	0.07	0.08	0.06	0.08	0.08	0.09
BMIA → SBP	**0.19**	0.15	**0.24**	**0.18**	0.19	**0.19**
EDU → SBP	**-0.05**	**-0.03**	**-0.06**	-0.00	0.02	-0.00
L_INC → SBP	**-0.04**	**-0.07**	**-0.04**	0.02	0.01	0.02
EXERC → DBP	0.03	-0.00	0.04	0.01	0.03	0.01
SMOKCAT → DBP	-0.01	-0.01	-0.03	-0.03	-0.01	-0.04
ALCQ → DBP	**0.16**	**0.20**	**0.16**	0.06	0.04	0.06
BMIA → DBP	**0.18**	**0.17**	**0.21**	**0.15**	**0.18**	**0.15**
EDU → DBP	**-0.02**	-0.017	**-0.03**	0.01	**0.02**	0.01
L_INC → DBP	-0.00	-0.02	-0.00	**0.02**	0.01	0.02
SMOKCAT → HR	**0.22**	**0.23**	**0.20**	**0.18**	**0.19**	**0.16**
EXERC → HR	-0.02	-0.02	-0.01	**-0.25**	**-0.24**	**-0.26**
EDU → HR	**-0.03**	-0.02	**-0.03**	-0.01	-0.00	-0.01
L_INC → HR	-0.03	-0.02	-0.02	-0.00	-0.00	-0.01
ALCQ → BMIA	-0.00	0.04	-0.01	0.02	-0.00	0.02
SMOKCAT → BMIA	**-0.23**	**-0.24**	**-0.25**	**-0.19**	**-0.15**	**-0.19**
EXERC → BMIA	**-0.13**	-0.09	**-0.1**	-0.05	-0.02	-0.04
EDU → BMIA	**0.02**	**0.03**	**0.02**	**0.03**	**0.03**	**0.03**
L_INC → BMIA	**0.02**	**0.02**	**0.02**	**0.03**	**0.02**	**0.02**
EDU → EXERC	**0.06**	**0.07**	**0.06**	**0.06**	**0.06**	**0.06**
L_INC → EXERC	-0.00	0.00	-0.00	-0.00	0.01	-0.00
EDU → SMOKCAT	**-0.04**	**-0.04**	**-0.04**	**-0.04**	**-0.04**	**-0.04**
L_INC → SMOKCAT	0.00	-0.00	0.00	**0.02**	**0.02**	**0.02**
EDU → ALCQ	0.01	-0.00	0.01	0.01	**0.01**	**0.01**
L_INC → ALCQ	**0.03**	**0.04**	**0.03**	**0.03**	**0.03**	**0.03**

Only structural coefficients are shown.
Statistically significant coefficients ($\alpha = 5\%$) are in bold.
